# Rheumatoid Arthritis, Serologic Status and Risk of Heart Failure: A National Cohort Study

**DOI:** 10.3390/healthcare14213603

**Published:** 2026-10-23

**Authors:** Seonyoung Kang, Kyungdo Han, Yeonghee Eun, Jinhyung Jung, Seulkee Lee, Hoon-Suk Cha, Jaejoon Lee, Seonghye Kim, Se Yun Kim, Jiho Park, Dong Wook Shin, Hyungjin Kim

**Affiliations:** 1 Department of Internal Medicine, Samsung Medical Center, Sungkyunkwan University School of Medicine, Seoul 06351, Republic of Korea; 2 Department of Statistics and Actuarial Science, Soongsil University, Seoul 06978, Republic of Korea; 3 Division of Rheumatology, Department of Internal Medicine, Kangbuk Samsung Hospital, Sungkyunkwan University School of Medicine, Seoul 03181, Republic of Korea; 4 Samsung Biomedical Research Institute, Sungkyunkwan University School of Medicine, Suwon 16419, Republic of Korea; 5 Department of Family Medicine, Samsung Medical Center, Sungkyunkwan University School of Medicine, Seoul 06351, Republic of Korea; 6 Department of Internal Medicine, Konkuk University Medical Center, Seoul 16419, Republic of Korea; 7 Department of Clinical Research Design and Evaluation, Samsung Advanced Institute for Health Science and Technology, Sungkyunkwan University, Seoul 06351, Republic of Korea; 8 Department of Medical Humanities, Samsung Medical Center, Sungkyunkwan University School of Medicine, Seoul 06351, Republic of Korea

**Keywords:** rheumatoid arthritis, heart failure, seropositive, seronegative

## Abstract

Background: A limited number of studies have explored the association between rheumatoid arthritis (RA) and heart failure (HF) based on HF subtype (ischemic and non-ischemic) and RA serostatus. Objective: This study aimed to examine the association between RA and incident HF, considering serostatus (seropositive RA[SPRA], seronegative RA[SNRA]) and HF subtype. Methods: This retrospective cohort utilized nationwide population data from the National Health Insurance Service (NHIS) of Korea. The study analyzed 46,975 RA patients (33,424 SPRA, 13,551 SNRA) diagnosed in 2010–2017, along with 140,925 matched non-RA controls. Results: The RA patients had a higher risk of overall HF (adjusted hazard ratio (aHR) 1.97, 95% confidence interval (CI) 1.68–2.32), and risk was more prominent for ischemic HF (aHR 2.24, 95% CI 1.86–2.71) than non-ischemic HF (aHR 1.36, 95% CI 0.97–1.90). Compared with controls, the risk of overall HF was increased in both SPRA (aHR 2.13, 95% CI 1.78–2.55) and SNRA (aHR 1.59, 95% CI 1.19–2.11), with a non-significant trend toward a higher risk in SPRA than in SNRA (aHR 1.35, 95% CI 0.99–1.83). For non-ischemic HF, the risk was increased in SPRA (aHR 1.60, 95% CI 1.12–2.28) but not in SNRA (aHR 0.71, 95% CI 0.32–1.51); however, this finding was based on a small number of events. Conclusions: This study demonstrates that RA was significantly associated with an increased risk of overall and ischemic HF, with a non-significant trend toward an increased risk of non-ischemic HF. The increased risk of ischemic HF was observed in both SPRA and SNRA, whereas the difference in non-ischemic HF risk by serostatus should be interpreted with caution due to the small number of events.

## 1. Introduction

Heart failure (HF) significantly impacts quality of life through a range of symptoms and exercise intolerance, increases healthcare costs, and leads to higher rates of complications and mortality. In patients with rheumatoid arthritis (RA), HF is clinically relevant not only due to these issues and limitations in RA treatment caused by certain RA medications related to cardiotoxicity [1] but also because it is associated with a higher mortality. A higher risk is observed not only compared to non-RA patients with HF but also compared to RA patients with ischemic heart disease (IHD) [2]. RA is a well-known risk factor for cardiovascular (CV) disease. Among these conditions, HF is the most common CV trait and has been associated with the highest number of death events, as well as earlier deaths [3].

There is accumulating evidence of an increased risk of HF associated with RA. A population-based study from Minnesota, USA, involving 757 patients with RA revealed a 1.87-fold higher risk of overall HF compared to age- and sex-matched non-RA patients in the control group after adjusting for CV risk factors [4]. Another hospital-based study from the USA involving ~10,000 RA patients reported a 1.21-fold higher risk of overall HF compared to patients without an autoimmune disease [5]. A Swedish rheumatology registry study reported a 1.22-fold higher risk of HF in RA patients, with similar estimates for ischemic and non-ischemic HF. This remains the largest assessment to date, involving 235,235 RA patients [6]. A Danish register study reported a 2.3-fold higher risk in the first year after diagnosis but a 1.4-fold higher risk after that [7]. A nationwide cohort study from the Republic of Korea that included only patients with seropositive RA (SPRA) reported a 2.5-fold higher risk than the non-RA group [8].

Despite the accumulation of evidence of these associations, several questions on this topic remain unresolved. First, the impact of seropositivity on RA-associated HF remains uncertain. No studies have distinguished SPRA and seronegative RA (SNRA) when evaluating the association between RA and the risk of HF, despite the clinical and pathophysiological differences between the two [9,10]. Although some studies have evaluated the impact of rheumatoid factor (RF) positivity on this association [4], the role of anti-cyclic citrullinated peptide (anti-CCP) antibodies, which are more specific for RA and potentially could be linked to greater inflammation and higher CV disease [11], has not been thoroughly examined. Also, despite evidence that the increased incidence of HF cannot be solely explained by IHD, differential associations between RA and subtypes of HF by etiology, i.e., ischemic versus non-ischemic, rarely have been studied [12]. To the best of our knowledge, only one study has distinguished the subtypes of HF [6]. Therefore, whether the risk of HF in RA differs according to RA serostatus and HF subtype remains unclear. To address this gap, we conducted a nationwide population-based cohort study in Korea, in which seropositivity was defined using a national registration program requiring documented RF or anti-CCP positivity. This study aimed to evaluate the risk and cumulative incidence of overall, ischemic, and non-ischemic HF according to RA status and serostatus.

## 2. Methods

### 2.1. Data Source and Settings

We conducted a nationwide, population-based cohort study utilizing data from the National Health Insurance Service (NHIS) of the Republic of Korea, a government-operated insurance system that provides mandatory health coverage to nearly the entire Korean population (97%; the remaining 3% is covered by medical aid) [13]. Additionally, the NHIS offers a biannual national health screening program for individuals aged 40 years and older. The NHIS database encompasses medical diagnoses coded according to the International Classification of Disease 10th Revision (ICD-10), healthcare utilization, and pharmacy records. It also collects health-related data such as anthropometric measurements, health behaviors, and laboratory test results through the health screening program [14]. This dataset has been widely employed in epidemiological research. Personal identifiers were removed to ensure confidentiality, eliminating the requirement for individual informed consent. The study protocol was approved by the Institutional Review Board of Samsung Medical Center (IRB File No. SMC 2022-06-14). With this NHIS cohort data, we conducted a retrospective analysis to assess the association between RA and risk of HF.

### 2.2. Study Participants

We identified participants aged 40 years or older who were first diagnosed with RA between 2010 and 2017, selecting matched participants in the control group at a 1:3 ratio based on age, sex, and index year. SPRA was defined by the ICD code M05 together with registration in the Rare and Intractable Disease (RID) program (V223). The RID program is a nationwide co-payment reduction program administered by the NHIS, under which registered patients with designated diseases receive substantial financial support for their medical costs. SPRA is eligible for RID registration, whereas SNRA is not. Registration as SPRA requires a diagnosis of RA by a rheumatologist, fulfillment of four or more of the seven ACR classification criteria, and positivity for RF or anti-CCP antibodies, with the supporting laboratory results submitted for approval. Therefore, all patients classified as having SPRA in this study had documented positivity for RF and/or anti-CCP antibodies. SNRA was defined by the ICD-10 code M06 (excluding M06.1 and M06.4) together with prescriptions for any DMARDs for at least 270 days (Table S1 in Supplementary Materials). A prior validation study demonstrated that RA could be accurately identified in the NHIS dataset using a combination of ICD codes and DMARD prescriptions, with a positive predictive value of 92.3% [15]. This method of definition has been utilized in previous studies [16,17,18,19].

Initially, 92,336 patients over the age of 40 with newly diagnosed RA were screened. From this cohort, 54,910 participants were included because they had undergone a health screening within 2 years prior to the index date to collect baseline covariate data and did not have other rheumatic diseases (as listed in Table S2 in Supplementary Materials). Patients with missing data on any of the variables of interest (*n* = 1925, 3.5%) and those with a history of HF before RA diagnosis (*n* = 3813) were excluded; thus, all analyses were performed as complete-case analyses. Those diagnosed with HF within 1 year after the index date (*n* = 372) were also excluded to reduce surveillance bias. Additionally, participants were matched by age and sex to a control group at a 1:3 ratio. Ultimately, 46,975 RA patients (33,424 with SPRA and 13,551 with SNRA) were included in the analysis.

**Figure 1:**
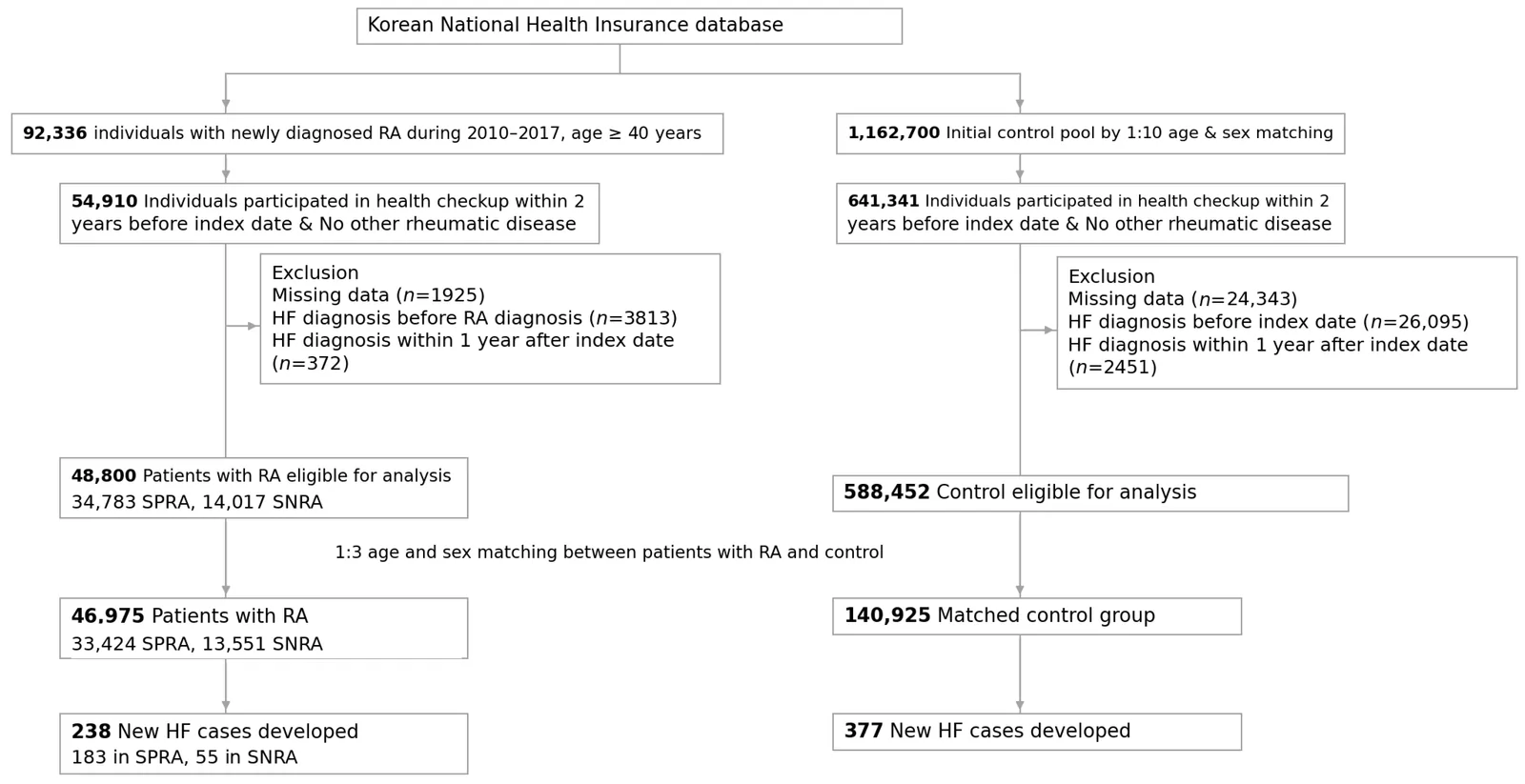
Flow diagram of study participants. Abbreviation: RA, rheumatoid arthritis; SPRA, seropositive RA; SNRA, seronegative RA; HF, heart failure.

For the control group, we applied the same inclusion and exclusion criteria. A total of 140,925 individuals without RA, matched 1:3 by age, sex, and index date to the RA patients, were included in the final analysis (Figure 1). The index date was defined as that of the first ICD code registration for SNRA patients and the first RID registration for SPRA patients. For the matched patients in the control group, the index date corresponded to that of the matched RA case. All participants were followed from 1 year after the index date until the occurrence of any censoring event, including the development of HF, death, emigration, or the end of the study period (31 December 2020). Unlike the Swedish and Danish studies, which tracked HF risk starting immediately from the index date and observed a strong RA–HF association within the first year [6,7], we applied a 1-year lag period to minimize reverse causation and surveillance bias, as pre-existing HF may be detected during the diagnostic work-up for RA. The same lag period has been applied in previous studies of RA using the NHIS database [16,17,18,19].

### 2.3. Outcome Definition

The primary outcome was new-onset HF, which was further classified into ischemic HF and non-ischemic HF. HF was defined as a primary diagnosis of HF (ICD-10 code I50) with at least one hospitalization, a definition that has been established and widely used in previous studies using the Korean NHIS database [20,21,22,23]. Requiring a primary diagnosis together with hospitalization, rather than any outpatient diagnosis, was intended to increase the specificity of the definition and to capture clinically significant HF events. HF subtypes were classified according to the presence of IHD (primary or secondary diagnosis of ICD-10 codes I20–I25) over the entire observation period, following a previous study [6]. Ischemic HF was defined as: (1) the first HF hospitalization in participants with an IIHD diagnosis at any time before, at the time of, or after the HF diagnosis, or (2) the first hospitalization with a primary diagnosis of ischemic cardiomyopathy (ICD-10 code I25.5). Non-ischemic HF was defined as a first-ever diagnosis of HF with hospitalization during follow-up in participants who had no history of IHD (primary or secondary diagnosis of ICD codes I20–I25) before or during the follow-up period [6].

### 2.4. Covariates

Comorbid conditions were defined using a combination of ICD-10 codes, medication prescriptions, and laboratory data. Sociodemographic factors, including alcohol consumption, smoking status, and physical activity, were collected from health screenings. Alcohol consumption was categorized as none (0 g/day), mild to moderate (<30 g/day for men; <20 g/day for women), and heavy (≥30 g/day for men; ≥20 g/day for women). Smoking status was classified as never, former, or current, and physical activity was categorized as active (vigorous exercise ≥ 3 days/week or moderate exercise ≥ 5 days/week) or inactive, following the American College of Sports Medicine guidelines [24,25]. Income was divided into the lowest 25th percentile (including medical aid) and higher percentiles. Body mass index was calculated and classified into five groups based on Asia-Pacific standards [26].

Other comorbidities were defined as (1) diabetes mellitus (DM) by ICD-10 codes (E11-14) with hypoglycemic medications or fasting glucose ≥ 126 mg/dL; (2) hypertension (HTN) by ICD-10 codes (I10-13, I15) with antihypertensive drugs or blood pressure ≥ 140/90 mmHg; (3) dyslipidemia by ICD-10 code (E78) with lipid-lowering agents or total cholesterol ≥ 240 mg/dL; (4) chronic kidney disease (CKD) by ICD-10 codes (N18-19) or eGFR < 60 mL/min/1.73 m^2^; (5) stroke by ICD-10 codes (I63, I64) with hospitalization and imaging; and (6) atrial fibrillation by ICD-10 code (I48).

### 2.5. Statistical Analysis

Baseline characteristics were described as means with standard deviations (SDs) for continuous variables and as frequencies with percentages for categorical variables. Comparisons of baseline characteristics according to RA status and seropositivity were performed using *t*-tests for continuous variables and chi-square tests for categorical variables. The incidence rates of HF were expressed as cases per 1000 person-years. The Kaplan–Meier method was employed to estimate the cumulative incidence of HF based on RA status and serologic classification. Multivariable Cox proportional hazards regression models were used to calculate adjusted hazard ratio (aHR) and 95% confidence interval (CI) for risk of HF. The models were progressively adjusted for potential confounders in three stages: (1) Model 1 adjusted for age, sex, income, smoking status, alcohol consumption, physical activity, and obesity; (2) Model 2 was further adjusted for DM, HTN, dyslipidemia, and CKD; and (3) Model 3 additionally adjusted for stroke and atrial fibrillation. Because of the small number of non-ischemic HF events in the SNRA group, we additionally performed an analysis adjusted only for age and sex. To assess the potential impact of unmeasured confounding, we calculated the E-value for the association between RA and overall HF. The proportional hazards assumption was assessed using Schoenfeld residuals. As sensitivity analyses, a competing risk analysis treating death as a competing event was performed using the Fine–Gray model, and subgroup analyses with interaction tests were conducted. A two-tailed *p*-value < 0.05 was considered statistically significant in all analyses, performed using SAS software version 9.4 (SAS Institute Inc. Cary, NC, USA).

## 3. Results

### 3.1. Baseline Characteristics

Table 1 presents the baseline characteristics of the study participants. Compared to participants without RA, patients with RA were more likely to be smokers, non-drinkers, and less obese. Patients with RA also had a higher prevalence of HTN, CKD, stroke, and atrial fibrillation than those without RA, whereas the prevalence of DM and dyslipidemia did not differ significantly between the two groups. Compared to patients with SNRA, those with SPRA were more likely to be older, female, current smokers, non-drinkers, and not obese. Comorbidities such as DM, HTN, dyslipidemia, CKD, and stroke were less common in patients with SPRA compared to those with SNRA.

### 3.2. Risk of Overall Heart Failure According to the Presence of RA and RA Serologic Status

**Figure 2:**
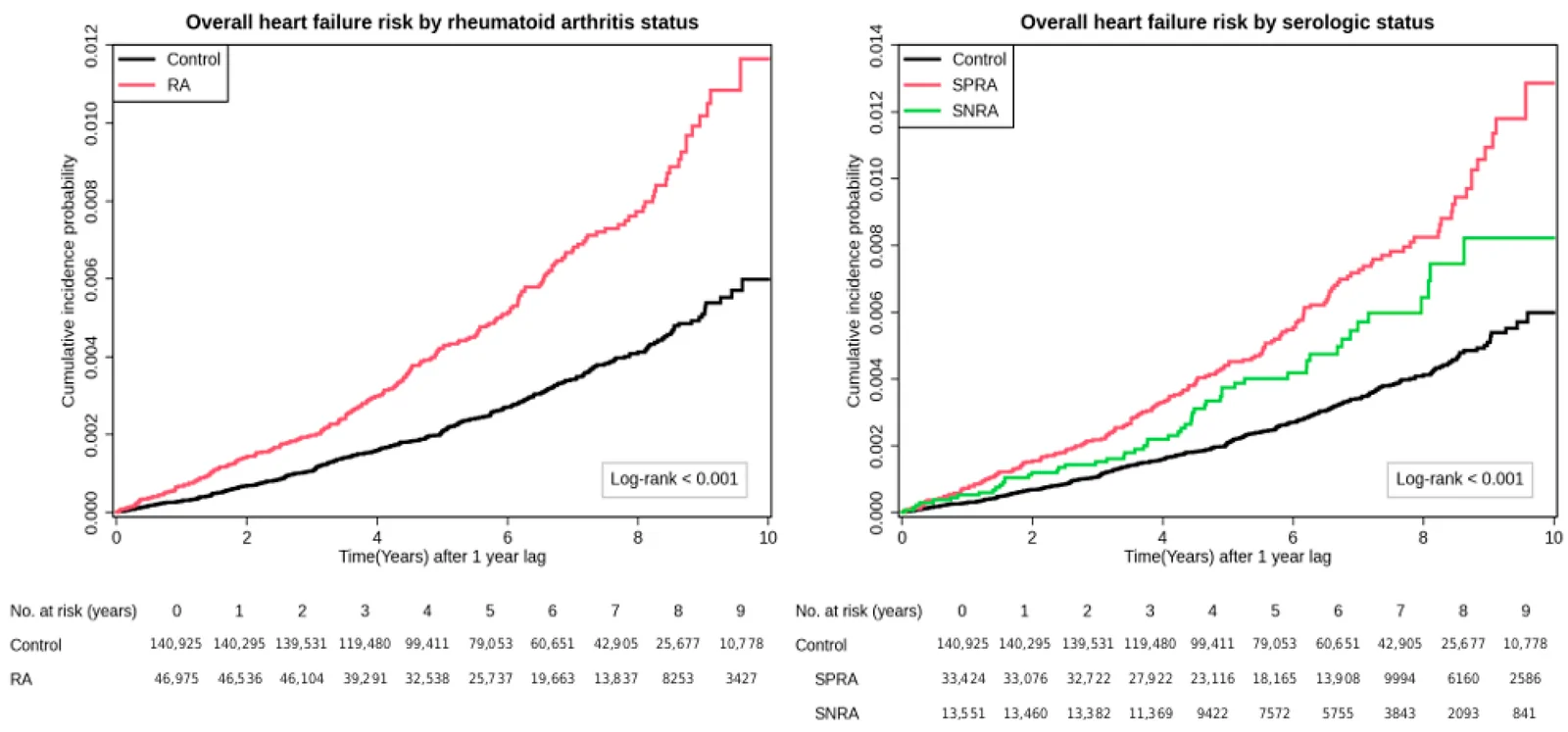
Cumulative incidence of overall heart failure associated with rheumatoid arthritis (RA) and serologic status of RA.

Over a median follow-up period of 5.46 years (interquartile range [IQR]: 3.68–7.45) with 1,048,335 person-years of follow-up after a 1-year lag period, we identified 615 new cases of overall HF (238/46,975 in the RA group, 377/140,925 in the control group). Patients with RA showed a 1.97-fold higher risk for overall HF than the control group (95% CI 1.68–2.32). Both patients with SPRA and SNRA were associated with an increased risk of overall HF compared to the control group (aHR 2.13, 95% CI 1.78–2.55 in SPRA; aHR 1.59, 95% CI 1.19–2.11 in SNRA). Patients with SPRA showed a non-significant trend toward a higher risk of overall HF compared to those with SNRA (aHR 1.35, 95% CI 0.99–1.83) (Table 2, Figure 2). The results were consistent in the competing risk analysis (RA vs. control: sub-distribution HR 1.86, 95% CI 1.58–2.19 for overall HF) and across most subgroups, except for a stronger association with ischemic HF in participants aged 40–59 years (*p* for interaction = 0.02) (Tables S3 and S4). The E-value for the association between RA and overall HF was 3.35 for the point estimate and 2.75 for the lower limit of the 95% CI.

### 3.3. Risk of Heart Failure Subtype According to Presence of RA and RA Serologic Status

Of the 615 overall HF cases, 451 were ischemic HF. During follow-up, 263 cases of ischemic HF occurred in the control group (of 140,925) and 188 cases occurred in the RA group (of 46,975). Patients with RA had a 2.24-fold higher risk of developing ischemic HF compared to the control group (95% CI 1.86–2.71). Patients with either SPRA or SNRA were associated with elevated risk of ischemic HF compared to the control group (aHR 2.37, 95% CI 1.93–2.92 in SPRA; aHR 1.94, 95% CI 1.42–2.64 in SNRA). The risk of ischemic HF was higher with SPRA than with SNRA, but the difference was not significant (aHR 1.21, 95% CI 0.87–1.69).

**Figure 3:**
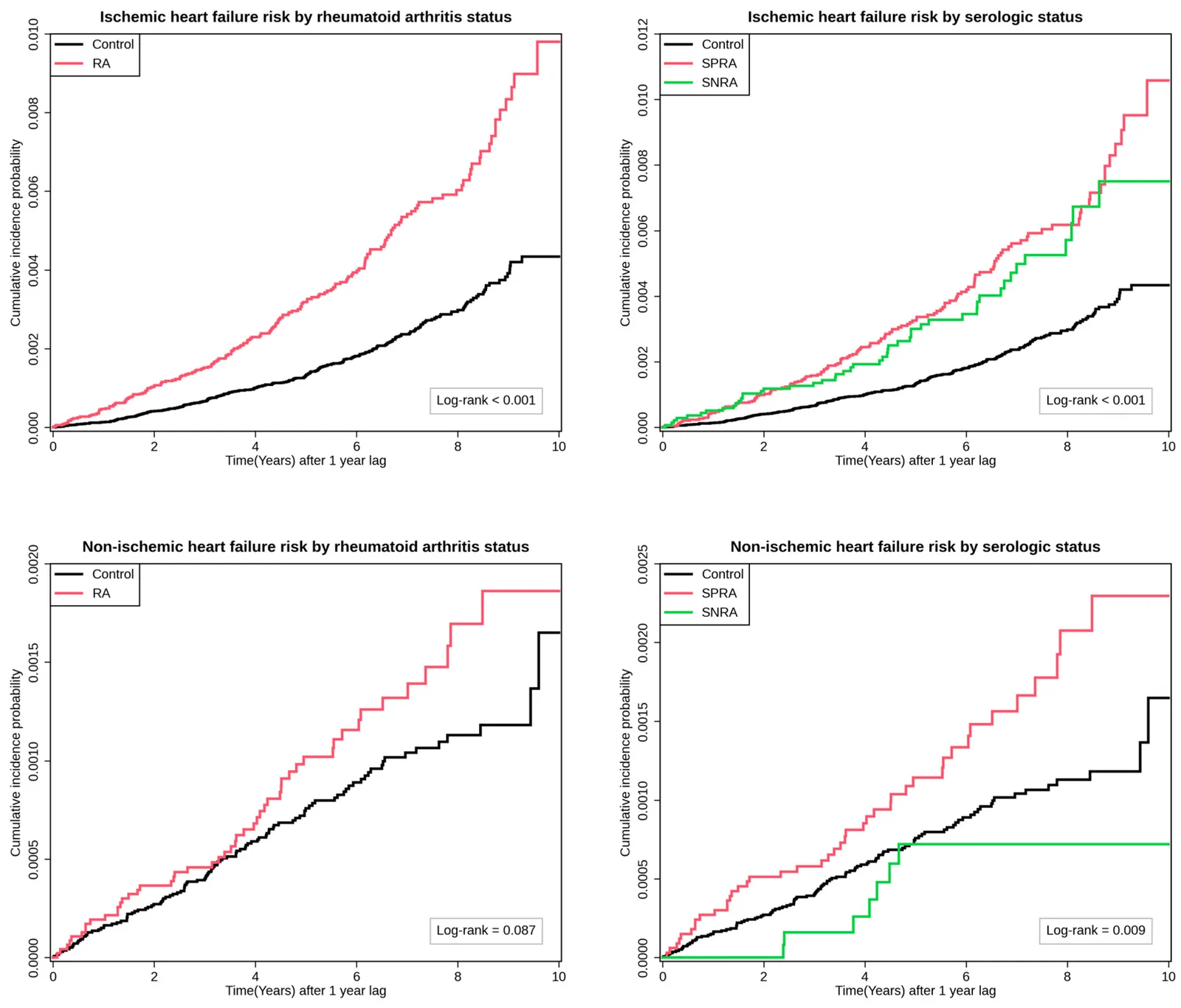
Cumulative incidence of heart failure subtype associated with rheumatoid arthritis (RA) and serologic status of RA.

A total of 164 cases of non-ischemic HF were identified, 114 in the control group and 50 in the RA group. Patients with RA showed a non-significant trend toward an increased risk of non-ischemic HF compared to the control group (aHR 1.36, 95% CI 0.97–1.90). Compared to the control group, patients with SPRA had an increased risk of non-ischemic HF (aHR 1.60, 95% CI 1.12–2.28), while patients with SNRA did not have an increased risk (aHR 0.71, 95% CI 0.32–1.51). Comparing patients with SPRA and SNRA, those with SPRA were associated with a higher risk of non-ischemic HF (aHR 2.31, 95% CI 1.03–5.17), although only seven non-ischemic HF events occurred in the SNRA group (Table 3, Figure 3). In models adjusted only for age and sex, the results were similar (SNRA vs. control: HR 0.68, 95% CI 0.32–1.47; SPRA vs. SNRA: HR 2.39, 95% CI 1.07–5.33) (Table S5).

## 4. Discussion

In this cohort study, our principal findings were as follows. First, RA was significantly associated with a higher risk of overall and ischemic HF, whereas only a non-significant trend toward an increased risk was observed for non-ischemic HF. Second, the risk of ischemic HF was increased in both SPRA and SNRA. The increased risk of non-ischemic HF appeared to be confined to SPRA; however, this finding should be interpreted with caution due to the small number of events.

### 4.1. Comparison with Previous Studies on RA-HF Association

We found that the risk of overall HF in RA patients was significantly higher compared to that of the non-RA group, which is consistent with previous studies [4,5,6,7]. A recent Korean study evaluated only SPRA and reported an aHR of 2.50 (95% CI 1.45–4.30) for SPRA-associated HF, which is similar to our results [8]. Most previous studies have treated HF as a single condition without detailed analysis of subtypes or underlying causes. Although the Danish and Swedish studies considered these factors, their methodologies differed from ours. For example, the Danish study assessed HF risk based only on the presence of IHD in patients with RA, which did not align fully with our study endpoints. Both studies tracked HF risk from the index date without applying a lag period, finding that HF risk was highest within the first year after RA diagnosis. They attributed this to increased inflammation in the early untreated stage, but it may also reflect higher HF detection due to increased healthcare utilization at the time of RA diagnosis. Furthermore, these studies defined RA using clinician diagnoses or ICD codes alone and included different age ranges than ours.

Due to these factors, direct comparisons are challenging. In addition, owing to differences in diagnostic criteria, treatment access, demographic structure, and genetic background, our findings may not be directly generalizable to other populations. Despite that, our study has several methodological strengths. Unlike prior studies, we used a validated RA definition that integrated ICD codes, RID codes, and long-term DMARD use to mitigate misclassification [4,6,7]. We also followed participants from 1 year after the index date to reduce surveillance bias and utilized a population-based design covering the entire Republic of Korea population, enhancing representativeness and minimizing loss to follow-up compared to previous hospital-based [5] or county-based studies [4]. Furthermore, we adjusted for key confounders, such as alcohol consumption [4,5,6,7,8], physical activity [4,5,6,7,8], and smoking [5,6,7,8], which are associated with both conditions and were not fully considered in previous studies.

### 4.2. Ischemic vs. Non-Ischemic HF

While RA was significantly associated with ischemic HF, only a non-significant trend was observed for non-ischemic HF. Few studies have evaluated the relationship between RA and HF in terms of ischemic versus non-ischemic HF [6,7]. However, our study differed from a Swedish study that reported a similar aHR for the two phenotypes of HF in RA (aHR 1.73 for ischemic HF and aHR 1.55 for non-ischemic HF) [6]. Nonetheless, our results can be understood in a similar context as Danish studies in which RA patients were divided into those with or without IHD and showed a higher risk of HF in RA patients with IHD (aHR 2.07) than those without (aHR 1.23) [7].

The primary pathogenesis of ischemic HF is driven by IHD, such as coronary artery disease (CAD), which is influenced by persistent inflammation, oxidative stress, and traditional cardiovascular risk factors. Inflammation is now recognized as a key factor in CAD, playing a role throughout all stages of disease development [27]. Stimuli such as dyslipidemia, HTN-related hormones, hyperglycemia byproducts, and pro-inflammatory cytokines induce endothelial activation, promoting leukocyte adhesion and migration into the arterial intima. This process is regulated by chemokines associated with both traditional and emerging risk factors, including RA. Once leukocytes infiltrate the intima, they interact with endothelial and smooth muscle cells, leading to cell proliferation and matrix formation, mediated by inflammatory components like cytokines. Oxidized lipids and advanced glycation end-products sustain this inflammation, contributing to plaque development and progression, ultimately impairing coronary circulation and leading to myocardial damage, characteristic of ischemic HF [28].

In contrast, non-ischemic HF is unrelated to CAD and is generally associated with structural and cellular changes in cardiac muscle that are not caused by atherosclerotic damage. Persistent inflammation is one of several mechanisms associated with non-ischemic HF, with chronic cytokine elevation leading to reduced cardiomyocyte contractility, hypertrophy, and ventricular remodeling [29]. Studies have shown a link between elevated inflammatory cytokines such as tumor necrosis factor (TNF)-α and IL-6, which are associated with RA, and myocardial pathology [30,31].

RA is a well-established independent risk factor for CAD, with mounting evidence supporting its causal role in vascular dysfunction, comparable to type 2 DM [32]. As discussed, atherosclerosis in RA may result from a combination of inflammation, oxidative stress, and traditional CV risk factors. RA may also influence CV risk profiles, increasing insulin resistance and altering adipokine levels, which interact with systemic inflammation to worsen vascular outcomes [33,34]. For example, HTN and DM in RA patients are associated with higher CAD event rates compared to non-RA patients [35]. Smoking is another overlapping risk factor that exacerbates both conditions, while RA-related pain and disability often lead to physical inactivity, which contributes to accelerated atherosclerosis [36]. Of particular importance, chronic systemic inflammation, which is a hallmark of RA, is a critical driver in the pathogenesis of CAD [37].

In RA-related non-ischemic HF, RA-cardiomyopathies may serve as a primary etiology, with notable manifestations including myocarditis, amyloidosis, and cardiac fibrosis. These complications are rare, and epidemiological data are limited, primarily derived from small case series and postmortem studies [37,38]. The occurrence of these complications is closely associated with greater disease activity, prolonged disease duration, and seropositive status [39]. This is partially supported by findings from a Swedish study, which highlighted a stronger association between RA disease activity and non-ischemic HF compared to ischemic HF [6]. Another potential explanation involves the correlation between anti-CCP antibody titer and degree of myocardial fibrosis [38].

Although the exact mechanisms underlying the higher risk of ischemic HF compared to non-ischemic HF in RA remain unclear, RA-related factors, such as pro-inflammatory cytokines, and traditional CV risk factors may have a greater impact on the development of CAD than on myocardial disease. Although the inflammatory response contributes to both ischemic and non-ischemic HF, it appears to be more important in vascular complications and only partially involved in myocardial disease, as discussed above. These insights carry important implications for clinical practice. Given the established link with CAD, preventive strategies that address traditional CV risk factors in RA patients are crucial. Additionally, effective RA management that reduces systemic inflammation is expected to be pivotal in preventing HF, irrespective of its subtype.

### 4.3. Serostatus

To the best of our knowledge, this is the first study to examine the risk of HF according to RA serostatus. We observed a non-significant trend toward a higher risk of overall HF in SPRA than in SNRA. Previous studies compared the HF risk by the presence of RF [6], or citrullinate antibodies [7,40], or disease activity level [6], but not by comprehensive diagnosis of seropositive or seronegative RA. For ischemic HF, the risk was similar with a 2-fold increased risk compared to that of non-RA controls for both SPRA and SNRA (aHR 2.4 and 1.9, respectively). This may be understood by the mechanism described above. Specifically, the shared RA-related inflammatory processes and CV risk factors contribute similarly to vascular disease in both conditions, suggesting serostatus itself may not directly influence this risk. Consistent with this, a nested case–control study in Sweden has found no association between RF and acute coronary syndrome risk, with only borderline significance observed for high levels of anti-CCP antibodies [41]. In contrast, the risk of non-ischemic HF appeared to be higher in SPRA than in SNRA. Given the small number of non-ischemic HF events in the SNRA group (*n* = 7), this finding should be interpreted with caution and regarded as hypothesis-generating. If confirmed, several potential mechanisms could explain this difference. First, the increased inflammatory burden related to severe disease activity in SPRA, despite ongoing debate, likely contributes to myocardial disease in RA, which is along the same lines as reports of RA-associated myopathies in cases of severe and long-standing RA. Second, the myocardium may serve as a potential autoimmune target in RA, especially in relation to anti-CCP antibodies. In non-ischemic HF, elevated levels of citrullinated proteins in the myocardium of RA patients can alter protein structure and function, serving as autoantigens for anti-CCP antibodies. This could trigger direct immune responses, leading to myocardial inflammation, interstitial fibrosis, myofilament injury, and remodeling [35,37,38], resembling the synovitis seen in RA [36]. Experimental studies have shown that myocardial citrullination is increased in HF patients, reducing myofilament calcium sensitivity and impairing contractility [36]. SNRA patients may be inherently less susceptible to the direct myocardial effects of anti-CCP antibodies, which could potentially account for the difference observed in our study. However, these hypotheses require further investigation and validation.

## 5. Limitations

There are several limitations to our study. First, residual confounding by unmeasured factors, such as RA disease activity and the use of DMARDs, glucocorticoids, and NSAIDs, cannot be excluded owing to the nature of claims data. Also, potential confounders such as genetic information and family history could not be accounted for due to data limitations. However, the E-value of 3.35 suggests that only a strong unmeasured confounder could fully explain the observed association. Second, we could not classify HF into commonly used ejection fraction categories (reduced vs. preserved) or account for baseline heart function because this information was unavailable. In addition, HF was identified using administrative data. Although the HF definition, restricted to a primary diagnosis with hospitalization, has been widely used in previous studies using claim data, it has not been formally validated in the Korean NHIS database; therefore, some misclassification of HF cannot be excluded. Third, our study might be subject to residual surveillance bias, although we applied a lag period to mitigate this issue. Sensitivity analyses using alternative lag periods could not be performed; however, the cumulative incidence curves diverged consistently after the 1-year lag. Fourth, the number of non-ischemic HF events in the SNRA group was small (*n* = 7). Therefore, the differences in non-ischemic HF risk by serostatus should be considered exploratory and require confirmation in larger studies. Additionally, because our study included participants who had undergone health checkups, there may be concerns regarding generalizability, as this group might represent individuals with healthier behaviors and better health status. Nevertheless, both RA patients and the control group underwent health screenings, limiting selection bias.

## 6. Conclusions

This cohort study found that RA was significantly associated with an increased risk of overall HF (aHR 1.97, 95% CI 1.68–2.32) and ischemic HF (aHR 2.24, 95% CI 1.86–2.71), with a non-significant trend toward an increased risk of non-ischemic HF (aHR 1.36, 95% CI 0.97–1.90). The risk of ischemic HF was elevated in both patients with SPRA (aHR 2.37, 95% CI 1.93–2.92) and SNRA (aHR 1.94, 95% CI 1.42–2.64), whereas the difference in non-ischemic HF risk by serostatus should be interpreted with caution due to the small number of events. Careful consideration of the increased risk of HF in patients with RA is essential for cardiovascular monitoring and management.

## Abbreviation

RA, rheumatoid arthritis; SPRA, seropositive RA; SNRA, seronegative RA; CVD, cardiovascular disease; HF, heart failure; DMARDs, disease modifying antirheumatic drugs; bDMARDs, biologic DMARDs; tsDMARDs, targeted synthetic DMARDs; TNF, tumor necrosis factor; IL, interleukin.

## Figures and Tables

**Table 1:** Baseline characteristics of study participants.

	TOTAL (*N* = 187,900)	Status of RA			Serologic Status of RA		
		No (*N* = 140,925)	RA (*N* = 46,975)	*p*-Value	SPRA (*N* = 33,424)	SNRA (*N* = 13,551)	*p*-Value
Sex							
Male	46,284 (24.63)	34,713 (24.63)	11,571 (24.63)	1	7994 (23.92)	3577 (26.40)	<0.0001
Female	141,616 (75.37)	106,212 (75.37)	35,404 (75.37)		25,430 (76.08)	9974 (73.60)	
Age, mean ± SD	57.68 ± 9.71	57.68 ± 9.71	57.68 ± 9.71	1	57.93 ± 9.69	57.04 ± 9.73	<0.0001
40–59	111,064 (59.11)	83,298 (59.11)	27,766 (59.11)	1	19,415 (58.09)	8351 (61.63)	<0.0001
≥60	76,836 (40.89)	57,627 (40.89)	19,209 (40.89)		14,009 (41.91)	5200 (38.37)	
Income				0.1944			0.2628
Low 25%	42,038 (22.37)	31,427 (22.3)	10,611 (22.59)		7596 (22.73)	3015 (22.25)	
Others	145,862 (77.7)	109,498 (77.7)	36,364 (77.5)		25,828 (77.3)	10,536 (77.8)	
Smoking				<0.0001			<0.0001
Never	149,817 (79.73)	112,992 (80.18)	36,825 (78.39)		26,241 (78.51)	10,584 (78.1)	
Former	18,140 (9.65)	13,217 (9.38)	4923 (10.48)		3265 (9.77)	1658 (12.24)	
Current	19,943 (10.61)	14,716 (10.44)	5227 (11.13)		3918 (11.72)	1309 (9.66)	
Drinking				<0.0001			0.0001
Non	134,534 (71.6)	98,969 (70.23)	35,565 (75.71)		25,484 (76.24)	10,081 (74.39)	
Mild to Moderate	45,738 (24.34)	35,840 (25.43)	9898 (21.07)		6892 (20.62)	3006 (22.18)	
Heavy	7628 (4.06)	6116 (4.34)	1512 (3.22)		1048 (3.14)	464 (3.42)	
Physical activity	38,148 (20.3)	29,421 (20.88)	8727 (18.58)	<0.0001	6058 (18.12)	2669 (19.7)	<0.0001
BMI				<0.0001			<0.0001
<18.5	5500 (2.93)	3685 (2.61)	1815 (3.86)		1356 (4.06)	459 (3.39)	
18.5–23	74,689 (39.75)	54,855 (38.92)	19,834 (42.22)		14,414 (43.12)	5420 (40)	
23–25	47,140 (25.09)	35,645 (25.29)	11,495 (24.47)		8173 (24.45)	3322 (24.51)	
25–30	53,719 (28.59)	41,408 (29.38)	12,311 (26.21)		8529 (25.52)	3782 (27.91)	
≥30	6852 (3.65)	5332 (3.78)	1520 (3.24)		952 (2.85)	568 (4.19)	
DM	22,785 (12.13)	17,178 (12.19)	5607 (11.94)	0.1452	3899 (11.67)	1708 (12.6)	0.0045
Hypertension	67,849 (36.11)	50,191 (35.62)	17,658 (37.59)	<0.0001	12,143 (36.33)	5515 (40.7)	<0.0001
Dyslipidemia	59,203 (31.51)	44,281 (31.42)	14,922 (31.77)	0.1644	10,154 (30.38)	4768 (35.19)	<0.0001
CKD	11,967 (6.37)	8529 (6.05)	3438 (7.32)	<0.0001	2319 (6.94)	1119 (8.26)	<0.0001
Stroke	986 (0.52)	622 (0.44)	364 (0.77)	<0.0001	237 (0.71)	127 (0.94)	0.0106
Atrial fibrillation	241 (0.13)	133 (0.09)	108 (0.23)	<0.0001	78 (0.23)	30 (0.22)	0.806

Abbreviations: SD, standard deviation; RA, rheumatoid arthritis; SPRA, seropositive RA; SNRA, seronegative RA; BMI, body mass index; DM, diabetes mellitus; CKD, chronic kidney disease.

**Table 2:** The risk of overall heart failure is according to rheumatoid arthritis (RA) status and serologic status of RA.

	Total (*N*)	Event (*N*)	F/U (PY)	IR			Hazard Ratio (95% CI)	
					Crude	Model 1	Model 2	Model 3
By RA status								
Control	140,925	377	789,181.3	0.48	1 (Ref.)	1 (Ref.)	1 (Ref.)	1 (Ref.)
RA	46,975	238	259,153.7	0.92	1.92 (1.64, 2.26)	2.03 (1.72, 2.39)	2.01 (1.71, 2.37)	1.97 (1.68, 2.32)
By RA status and seropositivity								
Control	140,925	377	789,181.3	0.48	1 (Ref.)	1 (Ref.)	1 (Ref.)	1 (Ref.)
SPRA	33,424	183	184,664.9	0.99	2.07 (1.74, 2.47)	2.18 (1.82, 2.60)	2.17 (1.82, 2.60)	2.13 (1.78, 2.55)
SNRA	13,551	55	74,488.7	0.74	1.56 (1.17, 2.07)	1.66 (1.25, 2.20)	1.61 (1.21, 2.14)	1.59 (1.19, 2.11)
By RA seropositivity								
SPRA	33,424	183	184,664.9	0.99	1.32 (0.98, 1.79)	1.31 (0.97, 1.77)	1.36 (1.01, 1.84)	1.35 (0.99, 1.83)
SNRA	13,551	55	74,488.7	0.74	1 (Ref.)	1 (Ref.)	1 (Ref.)	1 (Ref.)

Abbreviation: IR, incidence rate (per 1000/PY); F/U, follow-up duration; PY, person-years; aHR, adjusted hazard ratio; 95% CI, 95% confidence interval; RA, rheumatoid arthritis. Model 1 was adjusting age, sex, smoking, alcohol drinking, physical activity, income, and obesity; Model 2 was adjusted additionally for diabetes, hypertension, dyslipidemia and chronic kidney disease; and Model 3 was adjusted additionally for stroke and atrial fibrillation.

**Table 3:** The risk of heart failure subtype according to rheumatoid arthritis (RA) status and serologic status of RA.

	Total (*N*)	Event (*N*)	F/U (PY)	IR			Hazard Ratio (95% CI)	
					Crude	Model 1	Model 2	Model 3
Ischemic heart failure								
By RA status								
Control	140,925	263	789,181.3	0.33	1 (Ref.)	1 (Ref.)	1 (Ref.)	1 (Ref.)
RA	46,975	188	259,153.7	0.73	2.18 (1.81, 2.63)	2.32 (1.92, 2.80)	2.29 (1.90, 2.76)	2.24 (1.86, 2.71)
By RA status and seropositivity								
Control	140,925	263	789,181.3	0.33	1 (Ref.)	1 (Ref.)	1 (Ref.)	1 (Ref.)
SPRA	33,424	140	184,664.9	0.76	2.27 (1.85, 2.79)	2.42 (1.97, 2.98)	2.42 (1.97, 2.98)	2.37 (1.93, 2.92)
SNRA	13,551	48	74,488.8	0.64	1.96 (1.44, 2.66)	2.07 (1.52, 2.82)	1.97 (1.45, 2.69)	1.94 (1.42, 2.64)
By RA seropositivity								
SPRA	33,424	140	184,664.9	0.76	1.16 (0.83,1.61)	1.15 (0.83,1.60)	1.22 (0.87,1.70)	1.21 (0.87, 1.69)
SNRA	13,551	48	74,488.7	0.64	1 (Ref.)	1 (Ref.)	1 (Ref.)	1 (Ref.)
Non ischemic heart failure								
By RA status								
Control	140,925	114	789,181.3	0.14	1 (Ref.)	1 (Ref.)	1 (Ref.)	1 (Ref.)
RA	46,975	50	259,153.7	0.19	1.33 (0.95, 1.86)	1.37 (0.98, 1.92)	1.37 (0.98, 1.92)	1.36 (0.97, 1.90)
By RA status and seropositivity								
Control	140,925	114	789,181.3	0.14	1 (Ref.)	1 (Ref.)	1 (Ref.)	1 (Ref.)
SPRA	33,424	43	184,664.9	0.23	1.61 (1.13, 2.29)	1.63 (1.15, 2.32)	1.62 (1.14, 2.31)	1.60 (1.12, 2.28)
SNRA	13,551	7	74,488.7	0.09	0.65 (0.30, 1.39)	0.70 (0.33, 1.50)	0.70 (0.33, 1.52)	0.71 (0.32, 1.51)
By RA seropositivity								
SPRA	33,424	43	18,4664.9	0.23	2.47 (1.11, 5.49)	2.40 (1.08, 5.35)	2.33 (1.04, 5.20)	2.31 (1.03, 5.17)
SNRA	13,551	7	74,488.7	0.09	1 (Ref.)	1 (Ref.)	1 (Ref.)	1 (Ref.)

Abbreviation: F/U, follow-up duration; IR, incidence rate (per 1000/PY); PY, person-years; aHR, adjusted hazard ratio; 95% CI, 95% confidence interval; RA, rheumatoid arthritis. Model 1 was adjusting for age, sex, smoking, alcohol drinking, physical activity, income, and obesity; Model 2 was adjusted additionally for diabetes, hypertension, dyslipidemia and chronic kidney disease; and Model 3 was adjusted additionally for stroke and atrial fibrillation.

## Data Availability

The data used in this study are available from the Korean National Health Insurance Service (NHIS) upon reasonable request and with approval of the NHIS, but restrictions apply to the availability of these data, which were used under license for the current study and are therefore not publicly available.

## Supplementary Materials

The following supporting information can be downloaded at: https://www.mdpi.com/article/10.3390/healthcare14213603/s1, Table S1. Disease-modifying antirheumatic drugs (DMARDs) used to define patients newly diagnosed with RA; Table S2. The International Classification of Disease 10th revision (ICD-10) and Rare and Intractable Disease (RID) codes used for the exclusion of study participants; Table S3. Competing risk analysis of heart failure according to rheumatoid arthritis status and serologic status, treating death as a competing event; Table S4. Subgroup analyses of the risk of heart failure according to rheumatoid arthritis status and serologic status; Table S5. Risk of heart failure according to rheumatoid arthritis status and serologic status in models adjusted for age and sex only.
