# Supplementary material for: Rheumatoid Arthritis, Serologic Status and Risk of Heart Failure: A National Cohort Study
Source: Healthcare (Basel). 2026 Oct 23;14(21):3603. doi: 10.3390/healthcare14213603 (PMC13762635; doi:10.3390/healthcare14213603)
Supplement: Supplementary file 1 [file healthcare-14-03603-s001.zip › healthcare-4581393-supplementary.pdf]

Supplement Online Content

Table S1. Disease-modifying anti-rheumatic drugs (DMARDs) used to define patients newly diagnosed with RA

Table S2. The International Classification of Disease 10th revision (ICD-10) and Rare and Intractable Disease (RID) codes used for the exclusion of study participants

Table S3. Competing risk analysis of heart failure according to rheumatoid arthritis status and serologic status, treating death as a competing event

Table S4. Subgroup analyses of the risk of heart failure according to rheumatoid arthritis status and serologic status

Table S5. Risk of heart failure according to rheumatoid arthritis status and serologic status in models adjusted for age and sex only

Table S1. Disease-modifying anti-rheumatic drugs (DMARD) used to define patients newly diagnosed with rheumatoid arthritis

Category	Drugs
csDMARD	Methotrexate, Hydroxychloroquine, Leflunomide, Sulfasalazine, Tacrolimus, Cyclosporine, D-penicillamine, Bucillamine, Azathioprine, Minocycline, Mizoribine
bDMARD	Adalimumab, Etanercept, Infliximab, Golimumab, Rituximab, Abatacept, Tocilizumab
tsDMARD	Tofacitinib

csDMARD, conventional synthetic disease-modifying anti-rheumatic drug; bDMARD, biologic disease-modifying anti-rheumatic drug; tsDMARD, targeted synthetic disease-modifying anti-rheumatic drug

Table S2. The International Classification of Disease 10th revision (ICD-10) and Rare and Intractable Disease (RID) codes used for the exclusion of study participants

RID code	Disease
V111	Sarcoidosis
V133	Juvenile idiopathic arthritis
V134	Polyarteritis nodosa, Eosinophilic granulomatosis with polyangiitis, Juvenile polyarteritis
V135	Goodpasture's syndrome, Thrombotic thrombocytopenic purpura, Thrombotic microangiopathy, Granulomatosis with polyangiitis, Takayasu's arteritis
V136	Systemic lupus erythematosus
V137	Inflammatory myositis
V138	Systemic sclerosis
V139	Sjogren's syndrome, Mixed connective tissue disease, Behcet's disease, Polymyalgia rheumatic, Eosinophilc fasciitis, Multifocal fibrosclerosis, Weber-Christian disease
V140	Ankylosing spondylitis
V178	Relapsing polychondritis
V237	Psoriatic arthritis
V238	Microscopic polyangiitis
V290	Hypersensitivity vasculitis
V294	Cryoglobulinemic vasculitis
V298	Adult-onset Still's disease
V900	Extremely rare diseases including IgG4-related disease, SAPHO syndrome

ICD-10 code	Disease
M07	Psoriatic and enteropathic arthropathies
M31.0	Hypersensitivity angiitis
M45	Ankylosing spondylitis
D86	Sarcoidosis
D89.1	Cryoglobulinaemia
M06.1	Adult-onset Still's disease
M08	Juvenile arthritis
M30.0	Polyarteritis nodosa
M30.1	Polyarteritis with lung involvement (Churg-Strauss)
M30.2	Juvenile polyarteritis
M31.0	Hypersensitivity angiitis
M31.1	Thrombotic microangiopathy
M31.3	Wegener's granulomatosis
M31.4	Aortic arch syndrome (Takayasu)
M31.7	Microscopic polyangiitis
M32.1	Systemic lupus erythematosus with organ or system involvement
M33	Dermatopolymyositis
M34	Systemic sclerosis
M35.0	Sicca syndrome (Sjogren)
M35.1	Other overlap syndromes, mixed connective tissue disease
M35.2	Behcet's disease
M35.3	Polymyalgia rheumatica
M35.4	Diffuse eosinophilic fasciitis
M35.5	Multifocal fibrosclerosis
M35.6	Relapsing panniculitis (Weber-Christian)
M94.1	Relapsing polychondritis

Table S3. Competing risk analysis of heart failure according to rheumatoid arthritis status and serologic status, treating death as a competing event

	**Overall HF**	**Ischemic HF**	**Non-ischemic HF**
**By RA status**			
Control	1 (Ref.)	1 (Ref.)	1 (Ref.)
RA	1.86 (1.58–2.19)	2.10 (1.74–2.53)	1.30 (0.93–1.82)
**By RA status and serologic status**			
Control	1 (Ref.)	1 (Ref.)	1 (Ref.)
SPRA	1.97 (1.65–2.35)	2.17 (1.76–2.66)	1.51 (1.06–2.16)
SNRA	1.57 (1.18–2.08)	1.92 (1.41–2.61)	0.69 (0.32–1.49)
**By RA serologic status**			
SPRA	1.26 (0.93–1.71)	1.13 (0.81–1.57)	2.21 (1.00–4.87)
SNRA	1 (Ref.)	1 (Ref.)	1 (Ref.)

Values are subdistribution hazard ratios (95% confidence intervals) estimated using the Fine–Gray model, adjusted for age, sex, income, smoking status, alcohol consumption, physical activity, obesity, diabetes, hypertension, dyslipidemia, chronic kidney disease, stroke, and atrial fibrillation.

Abbreviations: HF, heart failure; RA, rheumatoid arthritis; SPRA, seropositive RA; SNRA, seronegative RA; Ref., reference.

Table S4. Subgroup analyses of the risk of heart failure according to rheumatoid arthritis status and serologic status

	By RA status		By RA serologic status		
**Subgroup**	**RA vs. control**	**P for interaction**	**SPRA vs. control**	**SNRA vs. control**	**P for interaction**
Overall heart failure					
Sex		0.397			0.357
Male	2.17 (1.65–2.86)		2.23 (1.64–3.02)	2.04 (1.33–3.14)	
Female	1.87 (1.53–2.30)		2.08 (1.67–2.59)	1.34 (0.92–1.96)	
Age, years		0.263			0.449
40–59	2.51 (1.60–3.95)		2.84 (1.76–4.60)	1.71 (0.77–3.83)	
≥60	1.90 (1.60–2.27)		2.04 (1.68–2.47)	1.58 (1.16–2.14)	
Income		0.181			0.283
Others	1.87 (1.56–2.24)		2.05 (1.68–2.50)	1.43 (1.03–1.98)	
Lowest 25%	2.47 (1.71–3.56)		2.51 (1.68–3.76)	2.36 (1.32–4.20)	
Current smoking		0.898			0.885
No	1.98 (1.66–2.36)		2.16 (1.78–2.61)	1.56 (1.15–2.12)	
Yes	1.92 (1.23–2.99)		1.97 (1.22–3.18)	1.76 (0.80–3.90)	
Alcohol consumption		0.881			0.794
No	1.99 (1.65–2.39)		2.12 (1.73–2.59)	1.66 (1.21–2.27)	
Yes	1.93 (1.34–2.76)		2.19 (1.48–3.23)	1.30 (0.65–2.59)	
Physical activity		0.212			0.121
Inactive	2.06 (1.73–2.46)		2.17 (1.78–2.63)	1.80 (1.34–2.42)	
Active	1.52 (0.98–2.37)		1.97 (1.25–3.11)	0.51 (0.16–1.64)	
Obesity		0.961			0.369
No	1.97 (1.60–2.42)		2.03 (1.62–2.55)	1.79 (1.26–2.54)	
Yes	1.98 (1.52–2.58)		2.31 (1.74–3.06)	1.28 (0.79–2.10)	
Diabetes		0.836			0.855
No	1.99 (1.65–2.42)		2.18 (1.77–2.68)	1.54 (1.09–2.17)	
Yes	1.92 (1.41–2.62)		2.01 (1.43–2.83)	1.70 (1.02–2.84)	
Hypertension		0.932			0.403
No	1.95 (1.44–2.64)		2.24 (1.63–3.07)	1.12 (0.58–2.14)	
Yes	1.98 (1.63–2.41)		2.08 (1.68–2.58)	1.75 (1.27–2.40)	
Dyslipidemia		0.954			0.808
No	1.98 (1.60–2.45)		2.08 (1.65–2.62)	1.70 (1.16–2.47)	
Yes	1.96 (1.52–2.53)		2.21 (1.67–2.91)	1.47 (0.95–2.26)	
Chronic kidney disease		0.942			0.719
No	1.98 (1.65–2.37)		2.16 (1.78–2.64)	1.51 (1.09–2.09)	
Yes	1.95 (1.35–2.82)		1.99 (1.31–3.01)	1.87 (1.05–3.32)	

Table S4. (continued)

Subgroup	By RA status		By RA serologic status		
	RA vs. control	P for interaction	SPRA vs. control	SNRA vs. control	P for interaction
Ischemic heart failure					
Sex		0.523			0.516
Male	2.43 (1.78–3.33)		2.45 (1.72–3.48)	2.41 (1.51–3.84)	
Female	2.14 (1.69–2.71)		2.33 (1.81–3.01)	1.67 (1.10–2.53)	
Age, years		0.020			0.063
40–59	4.57 (2.44–8.56)		4.67 (2.38–9.16)	4.33 (1.78–10.53)	
≥60	2.08 (1.71–2.54)		2.21 (1.77–2.75)	1.79 (1.28–2.50)	
Income		0.323			0.279
Others	2.15 (1.74–2.65)		2.33 (1.86–2.92)	1.72 (1.21–2.45)	
Lowest 25%	2.75 (1.76–4.28)		2.59 (1.57–4.28)	3.12 (1.64–5.93)	
Current smoking		0.855			0.661
No	2.23 (1.82–2.73)		2.39 (1.92–2.99)	1.85 (1.33–2.59)	
Yes	2.35 (1.39–3.96)		2.24 (1.26–3.99)	2.67 (1.18–6.05)	
Alcohol consumption		0.702			0.869
No	2.29 (1.85–2.82)		2.40 (1.90–3.02)	2.02 (1.44–2.84)	
Yes	2.09 (1.37–3.17)		2.29 (1.44–3.61)	1.63 (0.78–3.40)	
Physical activity		0.084			0.073
Inactive	2.40 (1.96–2.94)		2.45 (1.96–3.06)	2.30 (1.68–3.16)	
Active	1.46 (0.86–2.47)		2.03 (1.19–3.47)	0.23 (0.03–1.68)	
Obesity		0.652			0.149
No	2.32 (1.82–2.96)		2.29 (1.75–3.00)	2.41 (1.66–3.50)	
Yes	2.13 (1.58–2.87)		2.52 (1.82–3.47)	1.32 (0.75–2.30)	
Diabetes		0.566			0.756
No	2.32 (1.86–2.90)		2.48 (1.95–3.16)	1.93 (1.34–2.79)	
Yes	2.05 (1.43–2.94)		2.09 (1.40–3.13)	1.96 (1.11–3.46)	
Hypertension		0.777			0.813
No	2.35 (1.62–3.42)		2.56 (1.72–3.82)	1.73 (0.86–3.48)	
Yes	2.21 (1.78–2.75)		2.31 (1.81–2.94)	1.99 (1.41–2.81)	
Dyslipidemia		0.875			0.562
No	2.28 (1.76–2.94)		2.29 (1.73–3.04)	2.23 (1.47–3.39)	
Yes	2.21 (1.67–2.91)		2.47 (1.83–3.35)	1.68 (1.06–2.65)	
Chronic kidney disease		0.690			0.910
No	2.20 (1.78–2.72)		2.33 (1.85–2.94)	1.89 (1.33–2.68)	
Yes	2.42 (1.59–3.70)		2.55 (1.60–4.07)	2.15 (1.12–4.15)	

Table S4. (continued)

Subgroup	By RA status		By RA serologic status		
	RA vs. control	P for interaction	SPRA vs. control	SNRA vs. control	P for interaction
Non-ischemic heart failure					
Sex		0.682			0.816
Male	1.50 (0.84–2.68)		1.71 (0.92–3.19)	0.95 (0.29–3.11)	
Female	1.29 (0.86–1.95)		1.55 (1.01–2.39)	0.58 (0.21–1.61)	
Age, years		0.690			0.992
40–59	1.19 (0.57–2.48)		1.67 (0.80–3.48)	NE	
≥60	1.41 (0.97–2.05)		1.59 (1.06–2.38)	0.92 (0.42–1.99)	
Income		0.187			0.413
Others	1.19 (0.80–1.77)		1.40 (0.92–2.13)	0.63 (0.25–1.56)	
Lowest 25%	1.99 (1.04–3.79)		2.37 (1.20–4.67)	0.97 (0.22–4.11)	
Current smoking		0.707			0.984
No	1.39 (0.97–2.00)		1.62 (1.10–2.39)	0.81 (0.37–1.75)	
Yes	1.16 (0.48–2.80)		1.49 (0.62–3.58)	NE	
Alcohol consumption		0.659			0.744
No	1.30 (0.89–1.91)		1.51 (1.01–2.26)	0.75 (0.32–1.72)	
Yes	1.56 (0.77–3.16)		1.98 (0.95–4.10)	0.50 (0.06–3.72)	
Physical activity		0.564			0.645
Inactive	1.30 (0.90–1.88)		1.56 (1.06–2.30)	0.59 (0.24–1.46)	
Active	1.69 (0.75–3.76)		1.83 (0.76–4.40)	1.32 (0.30–5.71)	
Obesity		0.516			0.477
No	1.25 (0.83–1.90)		1.53 (0.99–2.36)	0.45 (0.14–1.45)	
Yes	1.58 (0.90–2.76)		1.75 (0.95–3.23)	1.18 (0.42–3.30)	
Diabetes		0.508			0.669
No	1.26 (0.84–1.89)		1.52 (1.00–2.31)	0.56 (0.20–1.53)	
Yes	1.61 (0.88–2.95)		1.83 (0.95–3.51)	1.07 (0.32–3.51)	
Hypertension		0.876			0.421
No	1.40 (0.83–2.35)		1.78 (1.05–3.01)	0.27 (0.03–1.95)	
Yes	1.33 (0.85–2.06)		1.48 (0.92–2.38)	0.96 (0.41–2.22)	
Dyslipidemia		0.385			0.748
No	1.48 (1.01–2.17)		1.73 (1.16–2.59)	0.75 (0.30–1.86)	
Yes	1.05 (0.52–2.08)		1.26 (0.60–2.65)	0.59 (0.14–2.49)	
Chronic kidney disease		0.405			0.188
No	1.45 (1.01–2.10)		1.79 (1.22–2.62)	0.52 (0.19–1.43)	
Yes	1.00 (0.44–2.24)		0.89 (0.33–2.35)	1.26 (0.37–4.20)	

Values are adjusted hazard ratios (95% confidence intervals). Models were adjusted for age, sex, income, smoking status, alcohol consumption, physical activity, obesity, diabetes, hypertension, dyslipidemia, chronic kidney disease, stroke, and atrial fibrillation. NE, not estimable because of the small number of events.

Abbreviations: RA, rheumatoid arthritis; SPRA, seropositive RA; SNRA, seronegative RA.

Table S5. Risk of heart failure according to rheumatoid arthritis status and serologic status in models adjusted for age and sex only

	Overall HF	Ischemic HF	Non-ischemic HF
**By RA status**			
Control	1 (Ref.)	1 (Ref.)	1 (Ref.)
RA	2.02 (1.71–2.37)	2.30 (1.91–2.78)	1.37 (0.98–1.92)
**By RA status and serologic status**			
Control	1 (Ref.)	1 (Ref.)	1 (Ref.)
SPRA	2.17 (1.81–2.58)	2.40 (1.95–2.95)	1.64 (1.16–2.33)
SNRA	1.64 (1.23–2.18)	2.05 (1.51–2.79)	0.68 (0.32–1.47)
**By RA serologic status**			
SPRA	1.31 (0.97–1.78)	1.16 (0.83–1.61)	2.39 (1.07–5.33)
SNRA	1 (Ref.)	1 (Ref.)	1 (Ref.)

Values are hazard ratios (95% confidence intervals) adjusted for age and sex.
Abbreviations: HF, heart failure; RA, rheumatoid arthritis; SPRA, seropositive RA; SNRA, seronegative RA; Ref., reference.
